# Co-creation of a mobile health program (MumCare) for preventing maternal cardiovascular disease after pregnancy complications

**DOI:** 10.3389/fgwh.2026.1717203

**Published:** 2026-04-30

**Authors:** Anne Cathrine Staff, Anja Dusik Sjaavik, Josef Noll, Meryam Sugulle, Bendik Fiskå, Atle Klovning, Mette-Elise Estensen, Ralf Dechend, Åsa Henning Waldum, Kari N. Solbrække, Gunvor Aasbø

**Affiliations:** 1 Division of Obstetrics and Gynaecology, Oslo University Hospital, Oslo, Norway; 2 Institute of Clinical Medicine, Faculty of Medicine, University of Oslo, Oslo, Norway; 3 Department of Technology Systems, University of Oslo; 4 Department of General Practice, Institute of Health and Society, Faculty of Medicine, University of Oslo, Oslo, Norway; 5 Department of Cardiology, Oslo University Hospital, Oslo, Norway; 6 ProCardio Centre of Innovation, Department of Cardiology, Rikshospitalet, Oslo University Hospital, Oslo, Norway; 7 Max-Delbrück-Center for Molecular Medicine in the Helmholtz Association, Berlin, Germany; 8 Helios Klinikum Berlin-Buch, Berlin, Germany; 9 Experimental and Clinical Research Center, A Cooperation Between the Max-Delbrück-Center for Molecular Medicine in the Helmholtz Association and the Charité - Universitätsmedizin Berlin, Berlin, Germany; 10 German Center for Cardiovascular Research, Berlin, Germany; 11 Department of Public Health Sciences and Interdisciplinary Health Sciences, University of Oslo, Oslo, Norway

**Keywords:** cardiovascular diseases, co-creation, patient participation, pregnancy complications, preventive health services, risk, self care, telemedicine

## Abstract

**Objective:**

Epidemiological studies show an increased risk for premature maternal cardiovascular disease in women after pregnancy complications, like preeclampsia, gestational hypertension and gestational diabetes mellitus. Our goal was to create a novel “digital companion” for women with such pregnancy complications, in the format of a mobile health-assisted user-centered follow-up software application (app).

**Methods:**

A cardiovascular postpartum follow-up program was developed as a digital companion, including a new mobile application (app), which is based on Norwegian obstetric and international guidelines. The MumCare app was developed through a co-creation process that included users, stakeholders, and clinical experts. Five qualitative interviews and 10 qualitative co-creative user testing interviews were conducted throughout the development stages to improve the perceived usefulness of the companion. The objective of the present study was to analyze the iterative co-creation process including users, stakeholders and clinical experts.

**Results:**

Phase 1 involved developing the companion within an interdisciplinary expert group through an iterative process in close dialogue with users. Explorative user interviews in Phase 2 (*n* = 5) supported the translation of guidelines into a structured app format, visualized as MumCare sketches for design, functionality and user communication. During Phase 3, the app sketches were revised in collaboration with users, in application interviews (*n* = 7). During Phase 4, the programmed prototype was refined through feedback from pilot users (*n* = 3). The user groups highlighted several app benefits, including a follow-up system of personal modifiable risk factors, a user-friendly system for tracking blood pressure over time, with individualized feedback and prompts. The use of non-ambiguous language and symbols was appreciated among users, who also contributed new content items to the app.

**Conclusion:**

User-centered co-creation improved several important features during the companion development process. The MumCare app is being tested in a prospective randomized controlled clinical trial that started in June 2024.

**Clinical trial registration:**

Clinicaltrials.gov reg., identifier NCT05835596.

## Background

Cardiovascular disease (CVD) is a leading cause of premature death and morbidity in women. Sex-specific causes of female CVD are under-investigated, and females are often under-represented in trials (1). Epidemiological studies show a strong association between female CVD and several pregnancy complications, including hypertensive disorders of pregnancy (HDP, including preeclampsia and gestational hypertension) and gestational diabetes mellitus (GDM) (2, 3). Pregnancy can thus be seen as a freely available, albeit clinically underexploited, cardiovascular stress test for women. Globally, CVD is the main cause of death for women, responsible for 35% of total deaths in women in 2019, and a major cause of disability (1). In 2019, 8.9 million women died from CVD and 275 million women were diagnosed with CVD (1). Prevention of CVD is therefore a global priority, as incidence and costs are increasing (4).

Myocardial infarction (MI) and stroke account together for the largest share of fatal CVD events in women. Primary prevention includes the prevention and reduction of risk factors leading to MI and stroke, including hypertension, a strategy likely to be most efficient when initiated early in life (5), in subclinical stages. Population-based studies from Denmark (6) and Norway (7) have shown that up to one-third of women develop hypertension within 10 years following a hypertensive disorder of pregnancy, the rates increasing with increasing age at delivery. Few women are, however, offered a systematic cardio-preventive follow-up after such pregnancy complications, whether giving birth in Norway or elsewhere, despite increasing international awareness over the last two decades (8).

**Figure 1:**
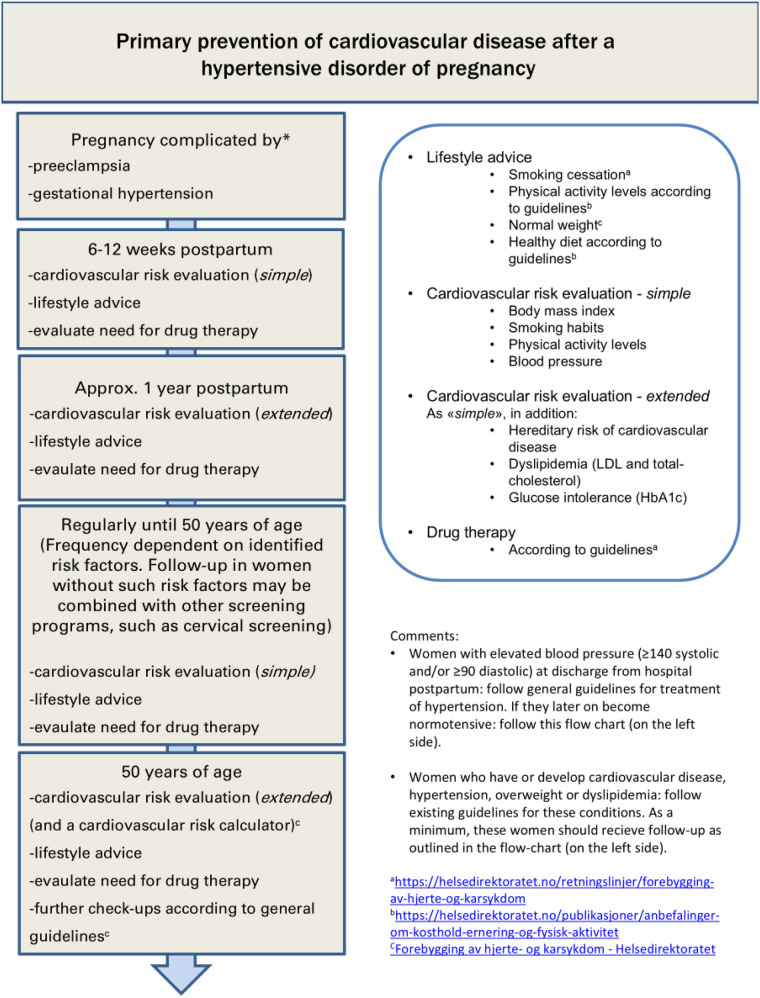
**(a)** Recommended follow-up in Norway after hypertensive pregnancy disorders (Norwegian web sited cited). **(b)** The 1a Figure is translated into a US-relevant web site setting in Figure 1b [previously published (49)].

Current international obstetric guidelines after adverse pregnancy complications like HDP and GDM have, until recently, not been very specific in proposing early preventive CVD strategies (3, 9, 10). These guidelines rely on expert opinions as randomized studies are lacking. European guidelines in cardiology suggest that an individualized cardio-preventive approach, including periodic screening for hypertension and diabetes mellitus following HDP and GDM, should be considered (11). Today, the national Norwegian obstetric guidelines (12, 13) suggest that women should be provided with information about (cardiovascular) CV follow-up recommendations after discharge from the hospital. Still, it is unclear whether this is implemented. A basic CV risk assessment following HDP or GDM is recommended at the patient's general practitioner at 3 months postpartum and at a 1-year follow-up in these guidelines (Figure 1a). At a 3–4 month follow-up, monitoring of blood pressure (BP) and body mass index (BMI) is recommended [as well as HbA1c (Hemoglobin A1c) for the GDM group].

**Figure:**
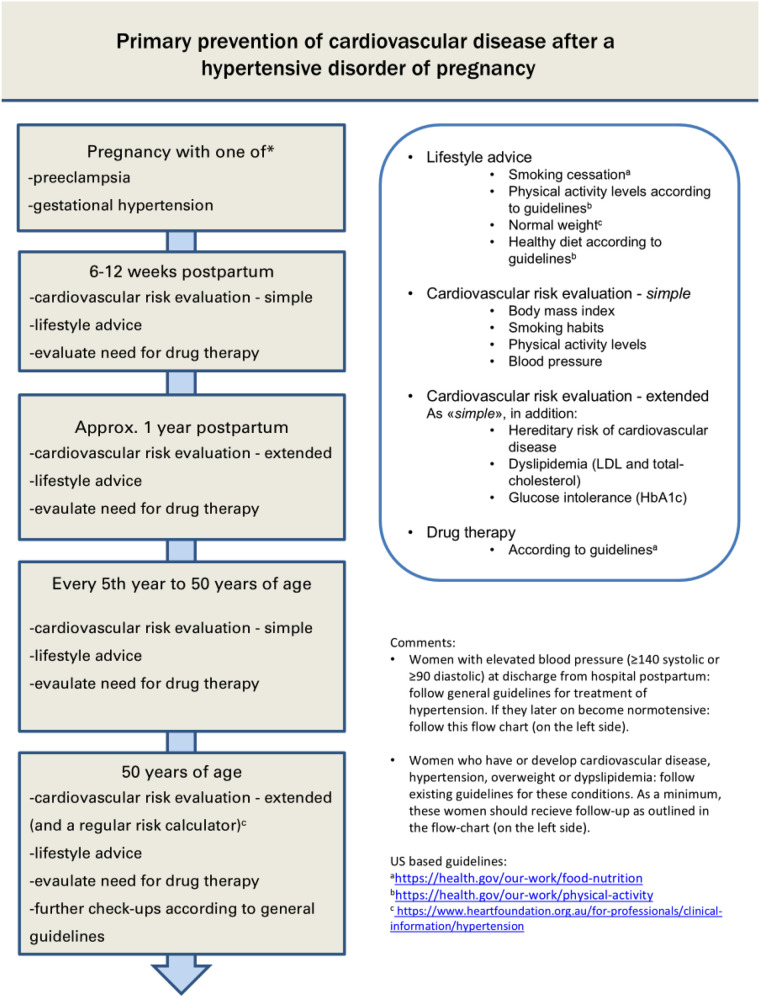


The 1-year postpartum assessment includes follow-up of BP, blood lipids, HbA1c, family history of CVD, BMI, smoking and alcohol history, physical activity and lifestyle (13). This follow-up is believed to facilitate continued personalized healthcare, addressing the specific risk factors for CVD identified at this visit. Hypertension and dyslipidemia are important modifiable CV risk factors for CVD, along with body weight. The Norwegian guidelines recommend individualized programs after the first postpartum year, but a minimum of BP monitoring every 3–5 years for those without other identified risk factors other than their pregnancy complication. This is based on the knowledge from both Norwegian (7) and Danish (6) studies showing that up to one third develop hypertension within the first 10 years after preeclampsia. Similarly, a recent study demonstrated that dyslipidemia already from a young age is strongly predictive of CVD (14).

Middle age is likely too late as a starting point for the most efficient reduction of modifiable risk factors and primary prevention of CVD. The risk of hypertension is high in the postpartum period after HDP (15, 16), and women with GDM may also have identifiable cardiovascular (CV) risk factors (17). The adverse pregnancy outcome could therefore be seen as a window of opportunity, where the postpartum period could be used for screening and targeting modifiable CV risk factors. Pregnancy represents a life-changing event, during which women are particularly focused on their own and their offspring's health. This setting may therefore represent an optimal time point to empower women to improve their own CV health for life, which is the focus of the MumCare digital companion. We propose that systematic preventive measures, starting postpartum with eHealth (electronic health)-supported programs, can improve individual maternal health literacy and CV health trajectories.

eHealth approaches have been suggested as measures to follow up women for long-term CV risk after HDP as well as GDM, including mHealth (mobile health)-assisted tools. Such eHealth tools targeting the postpartum period are, however, generally lacking today (18, 19). The World Health Organization (WHO) supports the potential of mHealth (17), emphasizing that “Digital health investments should be coordinated to support continuity of care” and that use of proactive reminder systems and mHealth technology warrants further research (20). Women's motivation for CV postpartum follow-up after a HDP pregnancy was recently explored in Norway, supporting women's interest in such follow-up (21). Women with a history of GDM wanted healthcare professionals to motivate them to make lifestyle changes (21). Some recent studies suggest that home blood pressure (BP) monitoring may be a useful screening modality in the detection and management of postpartum hypertension (22, 23). Comprehensive e- and mHealth assistance for health improvement postpartum are however lacking (3, 18).

Our MumCare study responds to these challenges by developing a new user-centered mHealth digital companion, intended to be introduced by clinicians during the patient's peripartum period. The companion includes a new app that enables the transition of patient care from specialist healthcare, focusing on common obstetric complications such as HDP and GDM, to general practitioners (GPs) within the primary healthcare system. GPs in Norway typically care for persons in need of primary prevention and CV follow-up. We constructed a postpartum follow-up app to benefit from the existing healthcare system in Norway, where a GP is assigned to every citizen. We designed the app addressing the health challenges of women recently identified with pregnancy complications associated with increased risk of future CVD, such as HDP and GDM. Our aim with the app is to improve women's preventive health literacy and to contribute to the optimization of women's CV health from a young age.

Co-creation describes a process where various stakeholders, including users, collaborate to develop a product. The advantage of co-creation is that the new ideas can be implemented to enhance the quality of the product. In the co-creation process there is a collaborative approach of creative problem solving between stakeholders at all stages of an initiative, from the problem identification and solution generation through to implementation and evaluation (24, 25). In developing eHealth resources, there is a widespread consensus in the eHealth research community that eliciting and addressing the needs and perspectives of the intended intervention user is a vital part of good intervention development (26).

This paper aims to detail the co-creation process involved in developing the mHealth MumCare companion, with a specific focus on the app, highlighting the roles and contributions of users, stakeholders, and interdisciplinary experts throughout each phase of the process. The main specific study questions were: 1) what characterizes the co-creation process of the MumCare digital companion? and 2) which co-creation mechanisms were most influential?

## Methods

### Study team, stakeholders and user involvement

The MumCare project PI (ACS) composed a project group in Oslo, Norway, consisting of an IT expert and scholar with experience in developing and testing an app for women during pregnancy after development of GDM (JN) (27), obstetricians (ACS, MS, BSF) and midwife (ÅW) with previous eHealth research experience (28), GP with experience from mHealth projects (AK), as well as two sociologists with expertise in user perspectives and qualitative research in women's health issues (GA, KNS). Two experienced cardiologists with expertise in CV diseases and pregnancy (MEE from Norway and RD from Germany) were also supporting the team, particularly in advising on BP feedback to the users. An important member of the team was a young medical student (ADS), an experienced health app user, who had a basic technological and medical education and particularly contributed to translating the app ideas into functional visual app features for young women.

Stakeholders from three nationwide expert organizations contributed during the early stages of project planning, lending support to the MumCare concept: The Norwegian College of General Practice (by leader Marte Kvittum Tangen), Norwegian Society of Gynecology and Obstetrics (by leader Ragnar K. Sande) and the Norwegian Society of Cardiology (by steering group member Eivind W. Aabel). These stakeholders and their networks provide important connections to Norwegian health policymakers, facilitating the future incorporation of MumCare study findings into revised guidelines and health services.

**Figure 2:**
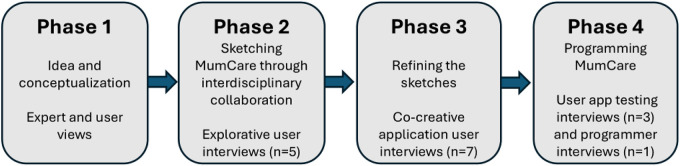
The 4 phases in the co-creative process of developing the MumCareapp.

The user representatives were from two nationwide patient associations, which assisted with patient information and recruitment, as well as the concept, design and testing of the MumCare app. This includes the Norwegian SIDS (sudden infant death syndrome) and Stillbirth Society (Landsforeningen uvented barnedød, LUB) (29), and the Society for adults with congenital or early onset of cardiac diseases (Voksne med medfødt hjertefeil), VMH) (30). LUB was formalized as a user representative support group from the planning and grant application period of the MumCare project. Both associations contributed important user perspectives in advising on communication with users in the app. Both LUB and VMH user representatives are also premenopausal women with postpartum experiences, easily identifying with the target group of the MumCare app. Both user representatives participated in a semi-structured interview (Figure 2; Phase 3). In addition to the participation of these user representatives throughout the entire co-creation process, further users were recruited for interviews and app testing, as described below in Phases 2–4 (Figure 2). These users were pregnant women diagnosed with HDP or GDM, hospitalized or treated as outpatients at Oslo University Hospital.

#### The mumCare app and IT security

The digital infrastructure, including the app and a supportive cloud infrastructure, is based on in-depth security and consent-driven privacy assessment. Core to the security and privacy design principles are encryption and de-identification of data. The University of Oslo, Department of Technology Systems (ITS), performed the security design and assessment. Besides two-factor authentication for the first start, the app will, at the woman's request, generate a one-time password enabling her GP to view home-generated health data (e.g., weight and BP trajectories), facilitating communication and control of such risk factors. The patient-generated app data is stored in the app itself. For the purpose of the ongoing MumCare Randomized Controlled Trial (RCT), the entered patient data is also stored at the specific server for sensitive data at the University of Oslo (TSD; Service for Sensitive Data), in agreement with the user's consent. This secure IT platform is designed for collecting, storing, analyzing, and sharing sensitive research data in accordance with Norwegian privacy regulations. Hospital-assembled health data will be stored at Oslo University Hospital, in accordance with both Norwegian and European laws and regulations, including the GDPR (General Data Protection Regulation). dHealth (a commercial health and self-management app developer) provided secure IT solutions and technical programming with several revisions of the MumCare app.

### A co-creation process in four phases

We employed a linear approach to co-creation, based on the Stage-Gate model (31). This is described as an innovation process model that divides the process into phases with quality checkpoints (“gates”) before each new phase can begin. During and after these phases, such quality check points were evaluated and discussed in project group meetings. In such meetings, the project group members solved potential challenges (e.g., revised a proposed app content or functionality based on new information from user interviews) and approved the transition to the next phase, if such challenges had been solved and tested. This approach to structuring the work ensured that development is based on thorough evaluations. The process alternated between internal planning and development within the project group and seeking feedback from users externally. This approach ensured a dynamic development that integrated both professional expert and user perspectives, resulting in an app that is both medically evidence based, technically solid and tailored to the users’ needs. The method in developing the MumCare app is illustrated as four Phases in Figure 2. Table 1 lists the educational items available in the English version of the MumCare app. Table 2 summarizes the qualitative interview content during the co-creation of the MumCare app, related to user involvement and the development phases.

**Table 1:** The educational items (all include relevant information and links to web sites and/or published papers links) as presented in the English version of the MumCare app.

**• Blood pressure**
○ 2 short MumCare videos (“How to measure blood pressure” and “What does the blood pressure imply?”) ○ Which symptoms during hypertension ○ Why is hypertension a health risk?
**• Pregnancy hypertension/preeclampsia**
○ Preeclampsia brochure for patients and health personnel ○ Preeclampsia ○ Pregnancy as a stress test for a mother's future health ○ Recommended follow-up after pregnancy complications
**• Diabetes in pregnancy (gestational diabetes)**
○ Gestational diabetes- brochure for patients and health personnel ○ Gestational diabetes ○ Pregnancy as a stress test for a mother's future health ○ Recommended follow-up after pregnancy complications
**• Healthy lifestyle**
○ Physical activity ○ Recommended weekly minutes with physical activity ○ Advice for healthy living ○ Tobacco cessation ○ Breastfeeding after pregnancy complications
**• Mental health after pregnancy complications**
○ Mental health after pregnancy complications ○ Hypertensive pregnancy disorders ○ Gestational diabetes mellitus ○ Mental health support following pregnancy complications
**• Partner information**
**• General practitioner information**

**Table 2:** Methods: qualitative interviews (by coauthors GA and ADS) performed, according to the MumCare app co-creation phases.

When	n	Who	Patient characteristics	Recruitment	Interviewer	Setting	Structure	Content
Phase 2 Spring 2023	5	Women hospitalised for observation for ongoing pregnancy complications [preeclampsia (*n* = 4) or gestational diabetes *n* = 1]	Four Norwegian born, one with immigrant background. Employment: communication, web design logistics, nurse 4 first pregnancy 1 third pregnancy	Research Midwife at Department of Obstetrics at Oslo University Hospital	GA	Patient room hospital	Semi-structured. Interview guide Duration 69 min (mean)	Explorative interviews: Motivation, risk perception and ideas of BP, app use, compliance and self-care
Phase 3 Fall 2023	2	Representatives from the user organisations	Norwegian born Employment: communication/ IT	Collaborative partners of the study: LUB^a^ and VMH^b^	ADS and GA	Hospital office/meeting room at the university	PowerPoint slides including sketches of the App as a starting point for dialogue and feedback. Duration 82 min (mean)	Application interviews: User experience, motivation and understanding Feedback on design, communication and functionality
Phase 3 Fall 2023	5	Outpatients with preeclampsia (*n* = 1)/gestational diabetes mellitus (*n* = 4)	3 Norwegian born 2 immigrant background 2; second pregnancy 3; first pregnancy	Research Midwife at Department of Obstetrics at Oslo University Hospital	ADS	3 at hospital, 1 at the work place of the patient, 1 digitally (zoom)	Design, communication and functionality Duration 64 min (mean)	App testing interviews: Revised sketches – user experience, motivation and understanding
Phase 4 Winter 2024/2025	a) b) c)	a) 2 outpatients with preeclampsia/gestational diabetes mellitus b) Representative from user organisation (VMH) with digital expertise c) Programmer (dHealth)	Norwegian born Norwegian born, working with IT Norwegian born, health app programmer	a) Research Midwife at Department of Obstetrics at Oslo University Hospital b) ADS c) GA/ADS	a) ADS b) ADS c) GA and ADS	Hospital Meeting room at work place Company meeting room	Duration 26 min (mean) Duration 45 min Duration 74 min	a) Prototype of the app-testing b) Prototype of the app-testing c) Development/programming process. d) Reflections, experiences

a Nationwide organization for bereaved families who have lost their child before or after birth.

b Society of adults with congenital or early onset of cardiac diseases.

### Phase 1: idea and concept development

#### Mumcare app study idea

The study idea was developed and discussed with the stakeholder collaborators, inspired by the increased focus on maternal long-term health after HDP and GDM over the last decade in the national guidelines (32, 33) and respective patient information (12, 13) from The Norwegian Society for Gynecology and Obstetrics. These long-term health aspects are in accordance with the preventive CV programs suggested in updated international expert guidelines (9, 10, 34–36), which the PI (ACS) have contributed to (9, 37). The guidelines from the Norwegian Directorate of Health for primary prevention of CVD after pregnancy complications (38) today mention indirectly, but do not link directly to, the obstetric guidelines for follow-up after preeclampsia (32), and do at present not mention HDP or GDM (33) as risk factors for CVD.

The MumCare app was planned to offer basic health-specific information and to assist in follow-up of modifiable CV risk factors in women with an increased risk of CVD after pregnancy. This aim aligns with the WHO's goals of increasing health literacy through digital health services (39).

Figure 1a illustrates the recommended follow-up after HDP in the Norwegian obstetric guidelines (13). For women after GDM, similar follow-up is recommended, complemented with an additional HbA1c control already at 3–4 months (12). Women with HDP or GDM are recommended to book a basic CV follow-up appointment at their GPs at 6–12 weeks postpartum as well as at 1 year postpartum. Women with an underlying medical condition in need of specialist care would be followed at the specialist care level. Also, if there are remaining hypertension or blood sugar irregularities before discharge from the delivery ward, the necessary additional postpartum follow-up visits are performed, either in specialist healthcare or in the primary health service, as appropriate. We planned the MumCare app to provide prompts for the users through the app, to book the recommended follow-up appointment at their GP, in line with these Norwegian national guidelines (12, 13). Figure 1b illustrates similar postpartum follow-up after HDP in a US-relevant setting, referencing publicly available US health information websites.

### Phase 2: explorative qualitative interview with users and multidisciplinary project group discussions

GA, ACS and KNS developed a semi-structured interview guide, aiming to generate insights into experiences related to diagnosis, reflection of current and future health risk, health apps use, information needs and future self-care. In order to explore the users’ different views and experiences regarding current pregnancy complications and future health risks and follow-up of their CV health, GA, a female sociologist in her 40s, conducted five such semi-structured explorative, qualitative interviews with pregnant women who were currently hospitalized with HDP or GDM. Further inclusion criteria were women with GDM or HDP, above 18 years of age and able to understand Norwegian and/or English, and were recruited through a research midwife at the Oslo University Hospital. Table 2 provides further details about the interviews conducted. At the time of the interviews the interviewed women were hospitalized for observation due to a pregnancy complication, and thus in a stressful situation. Nevertheless, all interviewees expressed that they enjoyed being interviewed. The interview was a break in an unpredictable and long day as hospitalized. GA made efforts to create a friendly and relaxed atmosphere for the interviews. GA emphasized before starting the interview that she was a sociologist, not a clinician and just keen to hear their experiences and reflections, also stating that their experiences and reflections could help to develop a better cardiovascular follow-up of women after complicated pregnancies. Interviews were audio recorded and transcribed, and safely stored at TSD, a platform holding the highest level of data security. The data was analyzed using the principles of reflexive, thematic analysis (40).

#### Co-creation within the multidisciplinary project group

**Figure 3:**
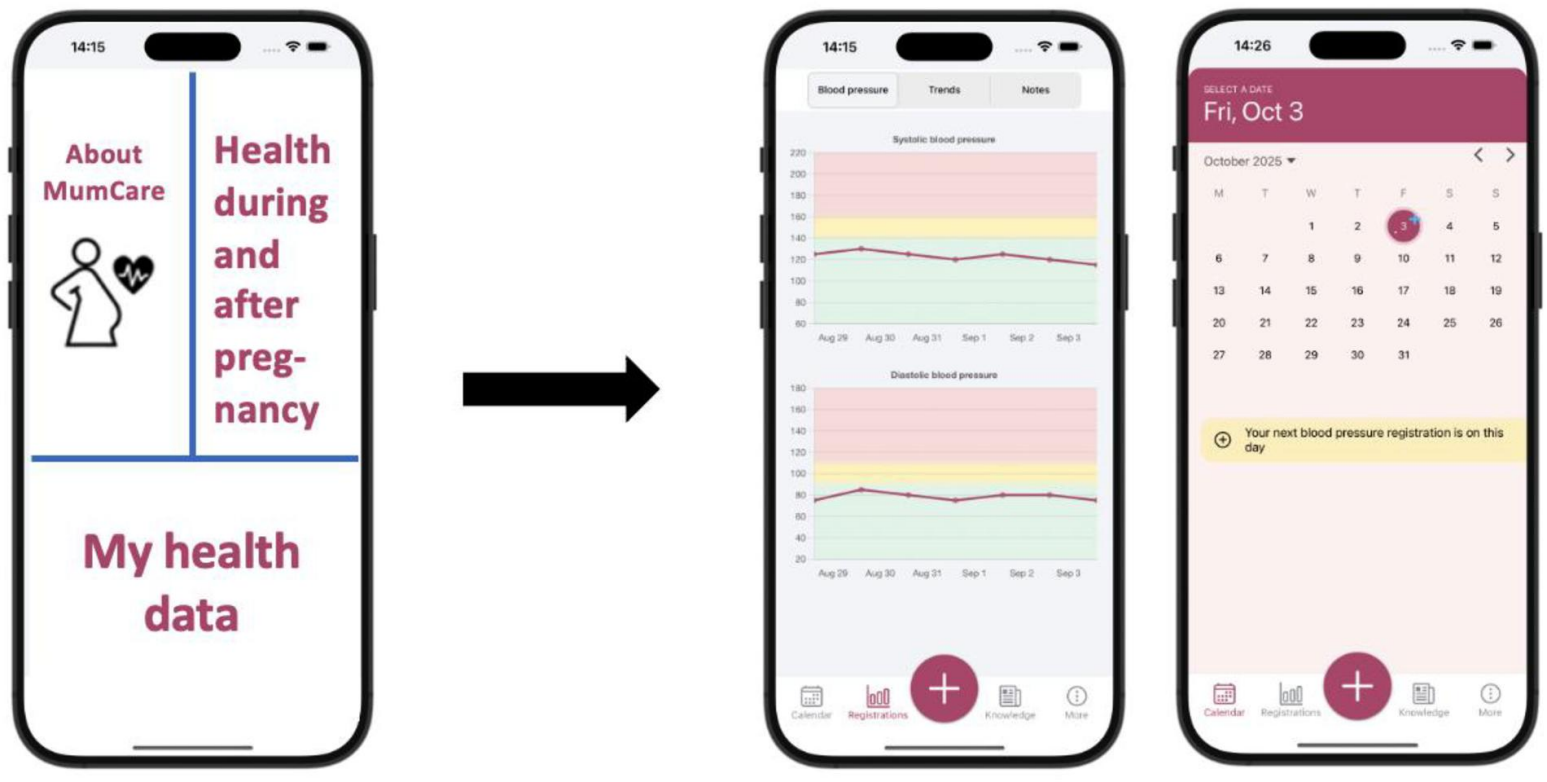
The MumCare app: from visual sketches to the programmed app. The left image of the first “page” of the MumCare app was shown to the testers in Phase 2-3. The right illustration shows the programmed first page of the MumCare app, as presented on the mobile phone (developed in Phase 4), where the first page presents a calendar with user reminders. The center image illustrates the visual representation of longitudinal self-registered blood pressures in the programmed app.

Based on the initial app ideas with inputs from expert groups, user organization representatives and users (patients), the project team finetuned the app content and developed visual app sketch proposals (AS, MS, GA and JN). In this initial phase, multiple ideas were explored, and various content was considered. The app's first “page” on the mobile phone was visualized using three parts, addressing the three main categories about, pregnancy health information and personal health data (see Figure 3).

The medical student (ADS), recruited to the project based on her excellent knowledge on health apps and their usage, developed the graphical ideas for the app's visual design, focusing on appealing and motivating features and design for the intended users, in close collaboration with the project group. The development of the MumCare app's visual design was inspired by popular apps for the relevant age group of young women (e.g., Strava, Lifesum, Clue, My Calendar, Visible, Fitbit, Duolingo). These apps are all characterized by having a simple and user-friendly way of conveying their message. A key feature of the MumCare app is built around the calendar (Figure 3) for tracking the registration of various health data over time (such as BP, weight, blood sugar, and physical activity), graphic visualization of registered data over time, and an educational/information channel.

The project group met regularly to discuss the design, including sketches, function and IT infrastructure/solutions as well as safety regards. In addition, the content of the educational part of the app was developed including both videos and text materials. Two short videos, featuring the PI (ACS), on “how to measure BP” and “what does high BP imply,” were created in both English and Norwegian. The four videos were also subtitled (in English or Norwegian) according to uniform availability principles.

**Figure 4:**
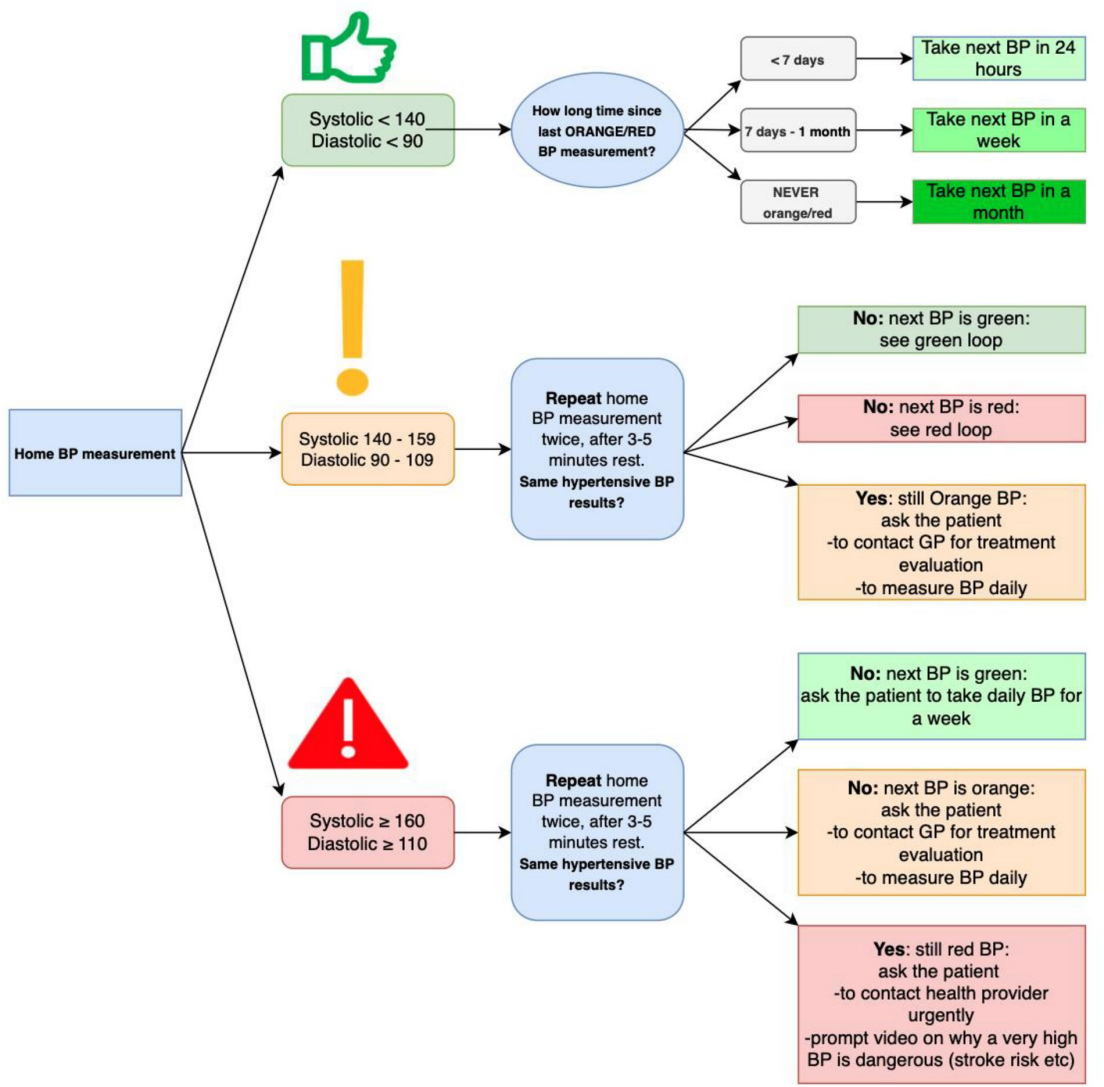
Feedback algorithm to the MumCare app user for blood pressure registrations entered into the app.

The MumCare app was designed to visually track the progression of patient-registered modifiable CV risk factors over time. It was planned to automatically provide BP feedback based on established national thresholds (see Figure 4). The app advises users to re-measure their BP after a five-minute rest period or to contact their GP if the readings exceeded specified limits.

A critical aspect of the app's development was incorporating user feedback on the design of notifications related to various BP thresholds. Additionally, the app would send push reminders to encourage women to schedule GP appointments at 3 and 12 months postpartum, aligning these reminders with their registered delivery date. This initiative aimed to bridge the gap between specialist healthcare during delivery and maternity care by GPs’ community health services.

Participants in the MumCare RCT, that was planned to start after the co-creation Phase 4, would receive a validated BP monitoring device suitable for use during pregnancy and in the postpartum period, following randomization to app access. The feedback algorithm, developed by ADS and AS (PI), employed a threshold of 140 mmHg systolic or 90 mmHg diastolic BP for hypertension, with severe hypertension defined as 160 mmHg systolic or 110 mmHg diastolic (Figure 4). These suggested thresholds and algorithms were validated by a team of healthcare experts, including cardiologists (MEE and RD), GPs (AK), obstetricians (MS, BF), and midwives (ÅW). It is important to note that the MumCare app is intended to function as a “digital companion” or “health coach”, and not designed, nor tested, to qualify as an approved medical device for formally diagnosing hypertension. The app's feedback serves as a warning for elevated BP, encouraging users to seek further medical diagnosis and care, if needed.

As part of the process of co-creating and discussing the app design, the project group prepared various sketches to obtain user feedback.

### Phase 3: application interviews with users and consecutive revisions of the app sketches

Further interviews with users were conducted by a female sociologist (GA) and a female medical student (ADS), including two interviews with representatives from the user organizations (LUB and VMH), four with women treated at Oslo University Hospital with ongoing GDM (including two women with a South Asian immigrant background to Norway), and one with a woman with preeclampsia. ADS conducted four of the patients interviews alone. Inclusion criteria were women with GDM or HDP, above 18 years of age and able to understand Norwegian and/or English. The interviewed pregnant women were recruited during admission to or at outpatient visits to the Department of Obstetrics at Oslo University Hospital. As a token of appreciation for their contribution to the research, participants received a universal gift voucher worth 500 kroner (42€). The interviews were focused on the app sketches. The interviews started out with a few introductory questions about pregnancy, health behaviour, as well as app use. The main part of the interviews included user-feedback on several animation slides that illustrated the app, showcasing various functionalities, design elements, communication features and educational resources. Table 2 provides further details about the interviews conducted. The app's visual design was consecutively changed according to the users’ feedback, and such changes or new alternative sketches were presented and discussed in the following user interviews. Before and throughout the interviews it was important to emphasize the value of user involvement and user feedback in the co-creative process of developing the MumCare app, actively welcoming and motivating the user's views and reflections. The team concluded that the user-feedback and comments had reached saturation when no new suggestions, comments or questions were posed in the last two interviews conducted. All interviews were audio recorded, transcribed and safely stored at TSD, a platform holding highest level of data security.

### Phase 4: co-creation through the app programming phase

The last phase of the co-creation process included app programming and testing. The multidisciplinary project group worked in collaboration with the company dHealth. An experienced programmer from dHealth met with the core app development group members (AS, JN, ADS) on a weekly basis over three months (February 2024-June 2024). Here, detailed programming issues were discussed and the project group provided further fine-tuning of content, functionalities and design (Figure 3). The educational part of the app was finalized (Table 1), listing links to relevant web information. Coauthors reviewed these pages for content and clarity. Three interviews, including user testing of the app, were conducted by ADS. This included two interviews with patients in the target group and the same user representative from VMH that had been interviewed in Phase 3. The pilot testing included task-based user testing [“Register your blood sugar/blood pressure” (with fictious or measured values), “Read the educational part related to your pregnancy complications”, and “Find your registrations”]. In addition, some questions were posed to invite for reflections about the user experience. All tasks were completed successfully by the users and indicated that the app functionality worked as intended and met the objectives.

The programmer was interviewed in February 2025 (by GA and ADS) after the MumCare app had been finalised, to explore his views of the co-creation process of the app.

#### Ethics statement

The MumCare study has been approved by the local hospital authorities (Oslo University Hospital, Division of Obstetrics and Gynaecology) and by the Regional Committee for Medical and Health Research Ethics (REK, ref. 478376), as well as by the Data Protection Officers at Oslo University Hospital. The Norwegian Agency for Shared Services in Education and Research reviewed the data protection of the study and approved the study (ref. 227676). All users and stakeholders interviewed provided informed consent to participate in the study interviews.

## Results

The processual results are described from each of the four phases below to provide a comprehensive presentation of the co-creation of the MumCare app.

### Phase 1: idea and concept development

The discussions with the stakeholders from the three nationwide expert organizations and the user representatives ensured support for the MumCare app development ideas in Phase 1. The MumCare app was from the onset planned to assist postpartum women in registering and tracking their own BP, weight, physical activity and laboratory analyses (e.g., fasting blood lipids and HbA1c), and to provide personalized feedback to motivate app use and self-care. The app was planned to prompt the user to attend a one-year postpartum follow-up at the user's GP, in line with the guidelines. Such a visit was seen by all stakeholders in Phase 1 as an important gateway to assess modifiable risk factors and to plan further health follow-up visit content and frequency, as well as to evaluate the need for health interventions.

The two user organization representatives provided informal feedback related to the app concept in this Phase 1, feeding into the app content development discussions.

### Phase 2: explorative qualitative interviews (*n* = 5)

The explorative qualitative interviews in Phase 2 supported the conclusions from the Phase 1 discussions regarding a perceived user need for tracking modifiable cardiovascular risk factors after HDP and GDM. The interviews also provided perspectives on self-care and need for information related to own cardiovascular health. The analysis of the explorative interview with women in the user group, pregnant women with an ongoing pregnancy complication, is represented through three themes, “Uncertainties and self-care related to hypertension during and after pregnancy”, “Information needs and engagement in personal health”, and “MumCare app – a more personalized, risk-based app of BP monitoring”. The key points related to these themes are displayed in Table 3, while examples of quotes supporting these three themes identified are listed in Table 4.

**Table 3:** Results: three themes identified from explorative user interviews (Phase 2; *n* = 5 interviews).

Theme 1: Uncertainties and self-care related to hypertension during and after pregnancy
**• Experience of pregnancy complication**
○ A stressful situation ○ High BP understood as a strain on the body ○ Not feeling ill and experiencing no symptoms, thus hard to fully take the health risks related to high BP seriously ○ Primarily concerned about the baby's health ○ Regarding pregnancy complication as a “personal failure”
**• Uncertainty about causes and self-care**
○ Difficult to understand what high BP “is” and why they had developed high BP ○ Stress understood as a possible cause for high BP ○ How may they influence their BP? Or what they were doing “wrong”?
**• (Over)weight, risks, and shame**
○ (Over)weight received renewed attention during pregnancy ○ High BP increased focus on body size, and weight ○ Weight: not a neutral measure even during pregnancy
**• Self-care after childbirth**
○ No mention of the importance of monitoring BP after pregnancy ○ Challenging to prioritize their own health after delivery ○ Focus on healthy nutrition/lifestyle to prevent later health problems

Theme 2: Information needs and engagement (including app usage) in personal health
**• Communication with clinicians**
○ Health risks related to high BP insufficiently communicated, and communicated differently in diverse contexts ○ Believed doctors were careful not to scare them ○ Advised not to stress/worry and not to google their condition, despite a great information need and that doctors knew patients search online
**• Perceived lack of information**
○ Informed about the diagnosis of hypertension, but little additional information about risks related to high BP during and after pregnancy ○ Uncertainty about whether they had received enough information ○ No information about recommended follow-up after pregnancy ○ Information is needed about what will happen next
**• Information sources and trust**
○ Information from specialized clinicians was highly valued ○ Search in trusted, public sources such as Norwegian Institute of Public Health and Norwegian Directorate of Health. ○ Looked for personal experiences/narratives to make sense of own illness experience and situation, e.g., in “due date groups” on Facebook

Theme 3: MumCare app – a more personalized, risk-based app of BP monitoring
**• Experiences towards health and pregnancy apps**
** **○ Experiences with fitness apps/pedometers such as Fitbit and pregnancy apps such as Clue, Preglife, Nørs, Contraction timer or childbirth courses) ** **○ Regular use of apps: integrated into everyday routines
**• Concerns about health/lifestyle app use**
** **○ Apps may reinforce a “bad conscience” when uable to follow up recommendations and not making “good enough” health choices ** **○ Risk of “appification” of life and health: Apps may overly control life and shape understandings of health and health risks
**• Concerns about BP monitoring**
** **○ Frequent BP measurements would increase stress and worry? ** **○ BP may become a negative and dominating focus? ** **○ Strain to face reminders of high-risk and a possibly unhealthy body, especially when trying hard to live healthy?
**• Emphasis on personalization in the MumCare app: motivation and empowerment**
** **○ Positive to the idea of an app to nudge them to monitor their *personal* health risk factors ** **○ The “personal risk” angle made the app feel more relevant and meaningful, and representing something different from a general health/lifestyle app. ** **○ Awareness of increased *personal* health risk following high BP during pregnancy leads to motivation and dedication for lifestyle changes ** **○ Monitoring their personal risk was seen as potentially empowering
**• App retention and adherence**
** **○ BP registration might be stressful at first, but over time routinized ** **○ Finding a balance in frequency was seen as important for long-term use ** **○ Will users stay motivated and dedicated to regular and long-term BP registration?
**• Specific and detailed suggestions on app functionality and design elements**
** **○ The app: intuitive, accessible, and easy to use: “as little hassle as possible” ** **○ Minimalist and simple design: simple confirmation that BP is okay, data presented in a tidy and clear manner: “no extra fuss.” ** **○ Customization – to choose how they want to use the app and which features they want the app to have/not have (weight registration, physical activity not mandatory). ** **○ Engagement: Fun to have statistics, to chart one's health, to see trends/history. ** **○ Not overly intrusive notifications in the app. ** **○ Positive towards an information hub in the app but not overwhelmingly much information. ** **○ Start using the MumCare app and BP reg. already during pregnancy.

**Table 4:** Results: supporting quotes from explorative user interviews (Phase 2; *n* = 5 interviews) related to the three major themes identified.

Theme 1: Uncertainties and self-care in the context of hypertension during and after pregnancy	
Participant 1	As soon as the placenta comes out, you return to normal and get rid of these symptoms, but still, high blood pressure—it has affected the body in some way. Even though it's supposed to go away afterwards, it's still a strain on the body while it's happening. So, I think about the time after the pregnancy, and I think more about trying to live even healthier now.
P.1	Since my dad had a heart attack, I think a little about—how are my blood vessels? And have they become narrower? What can I do to prevent it or stay healthy, while I also live a somewhat stressful or hectic life in everyday life? But I think maybe now I should focus a bit more on myself and try to incorporate it [into my daily routines]. But that's what I think now; whether I will actually manage that is another question.
P. 2	That was perhaps the first thing that struck me when we started talking about high blood pressure—that I felt embarrassed. A bit like… I wouldn't want to tell anyone else… I feel a bit like a failure. Yes, because I got sick and things like that. I wish there was a bit more information about it.
P. 2	So, I thought a bit more about it, and of course, you start to Google a bit and so on. But I feel that being in the hospital has really reassured me, and I get the information I need and can ask questions too. But of course, I’m worried. I feel reassured that the baby is doing well. Yes, but then I think more about my health after the pregnancy. In relation to high blood pressure and the kidneys and stuff like that.
P. 2	Here at the hospital, you get the facts in a way, whereas when you Google, it's more about experiences. I try to avoid forums (on social media) and things like that, but sometimes it's comforting to read about others’ experiences.
P. 3	I found it [the diagnosis of high blood pressure] a bit stressful. The doctor says that stressing about it doesn't help, but I feel that it's a natural reaction.
P. 4	For my part, I think that blood pressure isn't necessarily seen as something very dangerous in itself. There hasn't been much drama surrounding it… I mean, blood test results may be a bit more exciting than blood pressure. I don't know, it's like it's an “underdog.” … And it's also something about the fact that if it's not that dangerous, or it's not about cancer or brain tumors or strokes or among those “bad guys,” then I think it's easy to forget about it, since I have other things to think about now. Yes, but it's important, of course. And it's obviously an indicator of my health. And there are so many things it's linked to. It's not just connected to pregnancy complications. At least when you think about the follow-up, which you might be focusing on particularly. It's like, yes, it started with pregnancy complications, but as you say, there are also other heart and vascular health issues. And it's a symptom of quite a lot, which is maybe a lot more important.
P. 4	I believe mothers have a tendency to down prioritize [their own health], of course. At least that's how I feel right now.

Theme 2: Information needs and engagement (including app usage) in personal health	
P. 2	Yes, it worries me in a way; you’re a bit uncertain about whether it affects the baby… I mean, they say everything is fine, which is good. But yes, you start to overanalyze things a bit. I actually feel I haven't gotten very much information. No, you just get reactions like, “Oh, that's high blood pressure.”
P. 3	I told the doctor that I didn't feel I need a sick leave. Because as I said, I felt very well, so it was more that she wanted me to try to rest more. The doctor just said that this could develop into pregnancy complications. And she explained a bit about that, but I think she didn't want to go too much into it, to avoid making me [worried]… and then she said, “Don't Google this,” but of course, you do.
P. 3	I think maybe after being admitted yesterday, I understand it's serious, and I find it a little difficult to get information about what is… I would’ve liked a bit more information about what happens next.
P. 4	I imagine that the blood pressure number can also become something that you should maybe just see as an analytical thing. But it can also greatly impact mental health… like an extra number, like weight or BMI, as just indicators. Yes, I compare a lot because I, too, have a high BMI or high weight. Yes, so it's just one more thing. Yes, you really want to follow along and make the right choices and have better health. But then there is something about how realistic it is to keep track of it all the time. Yes, for your mental health, I don't quite know how a kind of app could be created that isn't very overwhelming.
P. 5	No, as I said, I’m not very fond of apps in general, but if there's something that helps my health and so on, then I think maybe it's smart. So, I understand that some people are doing it. It's very nice that some want to help those who might not be so good at remembering such things. Everything that concerns my health and my baby is important, so if that app wants to help me, then I say yes, okay, fine, I can participate in that kind of project.

Theme 3: MumCare app – a more personalized, risk-based app of BP monitoring	
P. 1	I would have liked to have information [information hub] like that. It doesn't have to be a lot, but just some information. Yes, so you’re aware of [the risks].
P. 2	I like to get information. Yes, but I also feel I can somehow filter it out, so that I’m not worrying about things too early. I like to be informed and have the possibility to be proactive.
P. 3	It must be very simple and intuitive, not too much clicking and hassle, you know… as simple as possible. I’m interested in understanding and think it's a bit fun to see statistics and trends. It may trigger me to keep track of the development over time, and if you include other elements like activity or diet as you mentioned—that's also fun to keep track of. So, I think it's important that it doesn't become too complicated… There's a balance here. If it becomes too much to fill out, you just won't bother.
P. 4	I think that an app that was solely for blood pressure and didn't consider anything else is a good idea. It would be good to keep it very simple. Yes, and that would maybe attract people like me, who don't want any extra fuss. Then you can keep it emotionally minimal. It's just the data, presented in a clear and organized way.
P. 4	It might vary a bit from person to person whether they want weight and physical activity. That's a bit vague, maybe? But weight is very related. I thought you could just have a completely simple sidebar menu where you only have blood pressure and weight, and then you can choose to remove weight, making it simple and just strip it down completely.
P. 4	I mean, I’m not a healthcare professional, and it's probably quite easy to just measure your blood pressure at home, but if you measure at home and get totally crazy numbers, you might want to have a kind of alert that says you should measure again or contact a doctor or emergency services ASAP.
P. 5	I think that if it becomes too much… I think keeping track of blood sugar and blood pressure is fine. Maybe heart rate and weight, and that's enough. Because that might be too much, but I also understand if you think more holistically—everything affects these values that we’re going to enter in the app. So, I think it might become overwhelming if you also have to write what you’ve eaten and how many steps you’ve taken.
P. 5	It's positive when you know that you have to register—it gives you a better overview than when you don't. You just think, “Yes, that's probably right,” but when you see the numbers you write down, you have a better overview, I think. But the downside is using the mobile so much all the time; there's too much technology.

### Phase 3: user application interviews: app sketches (*n* = 7)

The initial app sketches created in Phase 2 were revised and further developed to enhance aspects of design, user communication, and functionality (exemplified in Figure 3). The development of the app designs was shaped by input from both the project group and users during the application interview process in Phase 3 of the study. The sketches were adapted to the interviewed women's views, preferences, and needs, ensuring that the app was based on their real needs and expectations. The user feedback interviews carried out in Phase 3 directly shaped the development of the MumCare app and particularly contributed to a more user-friendly design, better addressing the specific health monitoring needs of relevant women during and after pregnancy. Table 5 summarizes the main interview findings, and how the input changed the MumCare app content, design and functionality.

**Table 5:** Results: qualitative application interviews in Phase 3 (*n* = 7): user input and consequent alterations to the app during the MumCare app co-creation process.

Visual design; alterations according to user input			
Visual MumCare app proposals	User evaluation	Visual MumCare app changes	User commment following app changes
Pink/red color palette	Well received		
BP Color coding - Green = normal - Red = elevated - Purple = severely elevated	Purple less alarming than red and not intuitive that it represents more danger than a red color	Color coding changed to a “traffic light” model - Green = normal - Yellow/orange = elevate - Red = severely elevated	Clearer and better conveying severity of a very high BP
Intuitive symbols introduced: -Bathroom scale for weight -Syringe for blood sugar -Heart for BP	Well received. The symbols support easy navigation and recognition Heart as a symbol for BP was suggested to be enlarged to highlight its importance	Heart for BP measurement was enlarged	Well recieved
Intuitive symbols/concepts introduced: Training	The word “training” may not be inclusive enough. “Physical activity” was preferred	“Training” replaced with “Physical activity”.	More inclusive and less sensitive.
Streak score at the top of the screen in the app (gamification feature: to be presented when the user had entered several items)	Streak made the app interface look cluttered No need for streak to motivate the person to use the app. The app itself is motivating enough.	Streak score removed	The app visual design appears cleaner, simpler and more functional

Communication; alterations according to user input			
Communication proposal	User evaluation	Visual MumCare app changes	User commment following app changes
A clear feedback mechanism for BP readings was developed	Appreciation of clearly communicated that BP is fine or normal. Women addressed an information need, posing questions such as “What is high BP really? What are the symptoms of high BP” In case of severe elevated BP, pop-up warning sign including a notification/question asking: If you have headache: Please see a medical doctor.	Added clear and accessible information in the information hub in the app about BP and symptoms Easy accessible and user-friendly information about BP and symptoms was included in the information hub	Well received Well received
The instructive text after elevated BP: “Contact healthcare”	Preferring clearer instruction of action required	Contact emergency services or General Practitioner	Well received
A generic thumbs-up after every registration. Intended to encourage continued app registration.	Misleading, suggesting “all is fine” even with higher BP	Revised feedback system (no thumbs up if elevated/high BP): -Numerical BP readings shown. -Orange alert for elevated BP. -Red warning triangle for severely elevated BP.	Improved communication of the severity of high BP values was well received

App functionality; alterations according to user input			
Functionality propsals	User evaluation	Visual MumCare app changes	User commment following app changes
Registration of physical activity/training, e.g., registration of time spent on physical activity pr week	Women typically use other apps for activity tracking (e.g., Strava). Would prefer if the app could be linked to the activity tracking in other apps. An estimate of time spent on physical activity per week is too diffuse. Physical activity registration is not the main functionality of the app.	Possibility to customize the app. Users can choose which parameters to track/see (weight, physical activity, blood sugar, etc.).	Well received
Registration of weight. Weight after birth and the future aimed ideal weight	Diverging opinions about whether or not the user should need to register an aimed ideal weight. This can be problematic for some women, and not all women wish to register or track body weight Consensus that weight registration may be optional. User chooses whether weight is visualized on the app alongside longitudinal BP data graphics (or not)	Possibility to customize the app. Users can choose which parameters to track/see (weight, physical activity, blood sugar, etc.).	Well received
Registration of blood sugar level after giving birth	Women with GDM in pregnancy tracked their blood sugar closely already during pregnancy. Women addressed that they did not have an app or any system for registration of their blood sugar values during pregnancy.	The functionality of the app was changed. Possible to start using the MumCare app already during pregnancy to track/register blood sugar values. App expanded to log and categorize measurements (fasting vs non-fasting blood sugar). Fasting and non-fasting blood sugar values are displayed in different colors. The app provides graphical visualization of BP and blood sugar over time. BP feedback algorithm is activated only after the user enters a delivery date. This BP feedback system is not used during pregnancy, as it is designed for postpartum home monitoring, not specialist antepartum care. Women required to register delivery to activate the BP feedback.	Well received
Registration of lipids, blood tests, etc.	Important to keep the app as simple as possible. Some questioned the value of such registration, since the GP also keep this records.	Kept as a functionality in the app, not main focus. Must be registered manually by the app user in the “comments” section, if applicable	Well received
Adherence support: Countdown on home screen showing days until next BP measurement is due	Push notifications regarded as helpful to remind to perform (and register) BP measurements. Countdown not so important	Women receive notification/reminders to monitor and register BP in the MumCare app. When women register BP, they will receive an immediate feedback about when to register next time (but no countdown messages)	Well received.

BP, Blood pressure.

### Phase 4: programming the mumCare app and user testing (*n* = 3)

Following the redesign of the MumCare app sketches on content, structure and visual design throughout the co-creation process in Phase 3, the dHealth programmer coded the MumCare app in Phase 4 accordingly. The finalized MumCare app prototype was iteratively refined through project meetings and testing, both by the project group members and 3 users. The user input and the programmer's views on the proceedings are displayed in Table 6. The final user testing supported the conclusion that the MumCare app functionality worked as intended and met the objectives.

**Table 6:** Results from Phase 4: programming the MumCare app by the dHealth programmer (*n* = 1) and user testing (*n* = 3).

Phase 4.1 The core ideas of the MumCare app were communicated to the programmer
• Purpose: easy registration and monitoring of blood pressure and other modifiable cardiovascular risk factors after pregnancy complications • Design goal: clear, intuitive interface for all users • A minimalistic and appealing design to signal credibility and to support the importance of BP monitoring ○ Calendar as central navigation hub (start screen) ○ User taps “+” to register: blood pressure, physical activity, weight, blood sugar (fasting and non-fasting, Notes (e.g., medications, lab values such as HbA1c, lipids)

Phase 4.2 Programmer's view on the co-creation MumCare process/product ahead of programming
• Project aim and app structure clear and well developed. Ideal starting point for efficient implementation and programming. • Crucial for the programmer to understand the purpose and aims of the app in order to also improve and optimize the design and functionality. • Aiming to develop a clean and professional design. An appealing app would motivate most women to start using the app. How the app interact with women should motivate most women to use the app.

Phase 4.3 User/expert feedback during the MumCare app programming phase (*n* = 3):
• Calendar view perceived as applicable and useful • Appreciation of flexible visualization of self-registered data • Improvements on font size adjustments, text revisions, feedback symbols refined

Phase 4.4 Final user-testing (*n* = 3)
The app was pilot-tested by users. As all tasks (including measuring and entering blood pressure levels) were completed successfully, a conclusion was drawn that app functionality worked as intended and met the objectives.

Phase 4.5 Programmer's view on the co-creation and collaboration with the project group, following finalization of the programming and final user testing
• Scheduled meetings with MumCare project group and programmer during the programming phase: Weekly meetings were ideal to ensure good progression • The composition and size of the project group (4 people) was good. Beneficial to collaborate closely with the persons who were in the position to take decisions (The MumCare principal investigator and core project group members). • A dynamic and flexible co-creation with the project/expert group: room for technical and creative refinement during development. • Integrated revisions from user testing and project group feedback into the programming • All programmer-driven changes discussed with MumCare team and expert group • Slightly adjustment of theme colors, text colors, and icons (alignment with implementation constraints and web standards)

## Discussion

We have described the co-creation process of developing the MumCare app, including contributions from experts, stakeholders, and user groups. Through this comprehensive process, we obtained a broad range of perspectives. This contributed to ensuring that the MumCare app was as inclusive, informative and functional as possible, in relation to intended use and users. The study group received valuable input during the co-creation process that not only altered the visual design elements of the app (e.g., font size, text revisions and feedback symbols) but also its functionality (e.g., possibility to include antenatal tracking of BP and blood sugars). This way of developing an mHealth app, using co-creation, was both time-saving and cost-effective, consistent with other studies (41).

The overall intention with the MumCare app innovation is to reduce the burden of women's long-term CV health challenges after pregnancy complications like HDP and GDM. The MumCare app was designed primarily to be used during the first 18 postpartum months following HDP and/or GDM, though not restricting further postpartum use. The app includes an educational section that offers audiovisual information on these pregnancy complications and their association with premature CVD. It also provides links to patient information brochures and websites summarizing preventive measures and follow-up according to the Norwegian obstetric guidelines for preeclampsia, gestational hypertension (32) and GDM (33), as well as their relevant patient information [available in Norwegian and English (12, 13)]. The app incorporates the Norwegian Directorate of Health's general recommendations for primary CVD prevention, including information on a healthy diet and physical activity (38). The English version of the app provides the same information as the Norwegian version, but links to further information on websites with health information in English (UK and US websites), if the Norwegian website does not provide an English version.

The relevant user groups highlighted several benefits of the novel MumCare app. During qualitative interviews they welcomed mHealth-assisted follow-up of personal CV risk factors. They highlighted the benefit of a follow-up system of personal modifiable risk factors, the user-friendly system for tracking BP over time, with individualized feedback and prompts. The use of non-ambiguous language and clear symbols was appreciated among users, who also suggested new content items to the app. They suggested several changes to the functions and visual elements of the app, both before and during the app's programming. We believe that the co-creation process with users and stakeholders through all stages of the app development contributed to a user-relevant first version of the MumCare app, which is now being tested in an RCT in Norway.

None of the interviewed user representatives questioned benefitting from automated registered BP or physical activity data into the app, such as through a wristband with built-in sensor or other devices, as used by us previously (42). A previous mHealth study has documented that important longitudinal data can be collected in a structured way through such systems (43). The strict risk assessment in the preparation phase of the MumCare app did however not allow any other application programming interface (API) during this first step of app development and testing in the RCT format, except from data being sent to the TSD at University of Oslo (see above), and therefore such automated registered data could not be included at present.

The user-centric design chosen in the MumCare companion is based on a participatory framework by Holst et al. (44) and Borgen et al. (27), two of our previous studies. The intensive co-creation process following the participatory framework has an implicit perceived usefulness. A more formal user evaluation of the mobile companion will be performed as part of the ongoing MumCare RCT, based on the Health IT Usability Evaluation Model (Health-ITUEM) (45), revised by Balderas-Diaz et al. (46).

A co-creation process is often named according to levels of involvement (47). Thin co-creation refers to limited forms of collaboration, where participants have influence on parts of the product or service. Thick co-creation describes a deeper form of collaboration where multiple stakeholders actively participate through the entire design and innovation process. The MumCare development process was heavily dependent on user and stakeholder input but aligns best with the “Thin co-creation” term, as the MumCare expert team were the initiators and main drivers of all development aspects.

The app sketches and the programmed app in Norwegian were only tested by women fluent in Norwegian, which may represent a cultural and socioeconomic bias in the app development. We have translated the app into an English version, which is to be used by women who are not proficient in Norwegian or who prefer an English app version over the Norwegian one. Still, some newly immigrated women to Norway may experience linguistic and cultural barriers to both the Norwegian and English app versions. As an example, we plan an Urdu version, as in a previous health app developed by one of the group members (JN) (25), aiming to reduce potential language barriers for ethnic groups with high rates of GDM and type 2 diabetes (e.g., South Asian descendants). The next phase after this app programming phase is to test the MumCare app in an RCT, which started in June 2024 and is expected finalized in 2027, including CV follow-up within 14–18 months postpartum.

We argue that there is undoubtedly a need for evidence-guided follow-up after pregnancy complications and that pregnancy is an easily available stress test for women's cardiovascular health. Better postpartum BP and weight control could likely improve maternal long-term health. Postpartum health care may also have transgenerational health benefits (3). We hope that the MumCare app may provide an opportunity to reinforce proposed postpartum CV follow-up programs and empower women to take care of their own health. A bonus is that the women, through the available educational information in the MumCare app, could contribute to educating the health services (e.g., their GP) regarding national and international recommended follow-up after pregnancy complications. An additional health benefit of a postpartum app aiming at optimizing female CV health may be a reduced risk of adverse pregnancy outcomes in the subsequent pregnancy.

The MumCare app is available on the App Store and Google Play. Still, its content cannot be accessed without a personal study code, which is only available to women randomized to app testing postpartum in the ongoing RCT.

We will likely benefit from the close collaboration with user groups and stakeholders in further improving national guidelines for CV follow-up after pregnancy complications, following the MumCare study conclusion. These stakeholders and their networks provide important user links to Norwegian national health policy makers, facilitating the incorporation of MumCare study findings into revised guidelines and health services. We also envisage revising the MumCare app following feedback from users after the finalization of the ongoing postpartum RCT and providing free access to the app for women in need in the future. The MumCare group also aims to, following the RCT in Norway, develop a version of the MumCare app adapted to low-resource settings with limited access to routine postpartum healthcare.

Retaining participants is a major concern in mHealth studies (48). Based on the feedback from our user interviews, we excluded any elements of gamification from the MumCare app. The ongoing RCT will show whether the motivation of the included patients is sufficient to continue using the app through the first postpartum year. Whether a postpartum app, such as the MumCare app, is also perceived as relevant and helpful beyond the first 14–18 months postpartum as a “digital health companion” could be tested in a future study.

## Conclusion

We ensured acceptability of the MumCare app concept among experts and user organizations representatives in Phase 1 and from relevant users (women hospitalized with relevant pregnancy complications) in Phase 2 of the MumCare co-creation process. In Phase 3, users gave feedback on a sketched app design, communication aspects and functionality. In Phase 4, users tested the programmed app prototype and ensured user functionality. Whilst the concept development in Phase 1 was largely expert-driven, we evaluate the input from users and user group members during all development phases as instrumental for the final app design, content and functionality. We view Phase 1 as a crucial success factor for the project being brought forward. Without strong support for the postpartum MumCare concept from two relevant nationwide patient associations and three nationwide medical expert organizations, the motivation for and funding of an app development would have been difficult to identify.

## Data Availability

The original contributions presented in the study are included in the article/Supplementary Material, further inquiries can be directed to the corresponding author.
